# Supplementary figures and images for: The geometry of efficient codes: How rate-distortion trade-offs distort the latent representations of generative models
Source: PLoS Comput Biol. 2025 May 12;21(5):e1012952. doi: 10.1371/journal.pcbi.1012952 (PMC12068621; doi:10.1371/journal.pcbi.1012952)

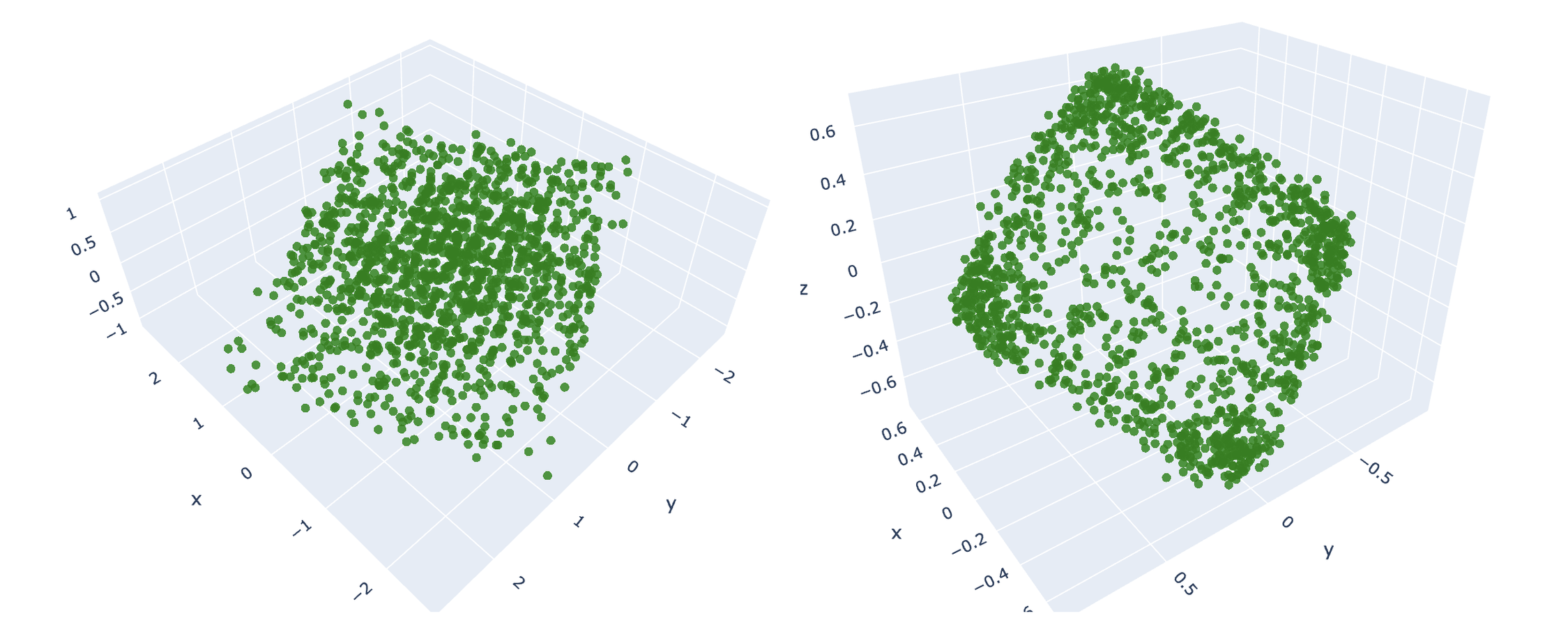

Supplement: S1 Fig — Comparison of the latent representations of the baseline model, trained at high capacity (left) and low capacity (right), in three dimensions (TIFF) [file pcbi.1012952.s001.tif]

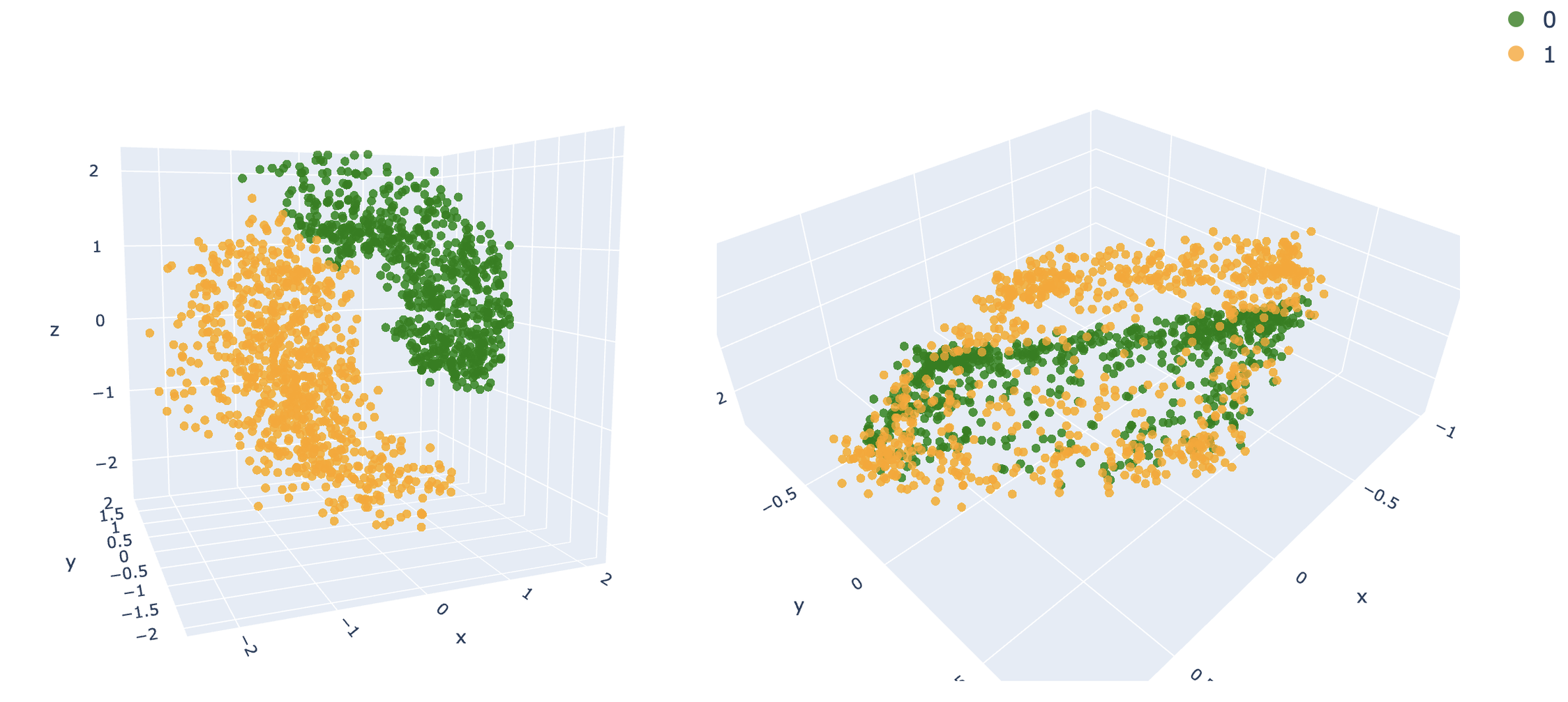

Supplement: S2 Fig — Comparison of the latent representations of the model E1M1, trained at high capacity (left) and low capacity (right), in three dimensions. (TIFF) [file pcbi.1012952.s002.tif]

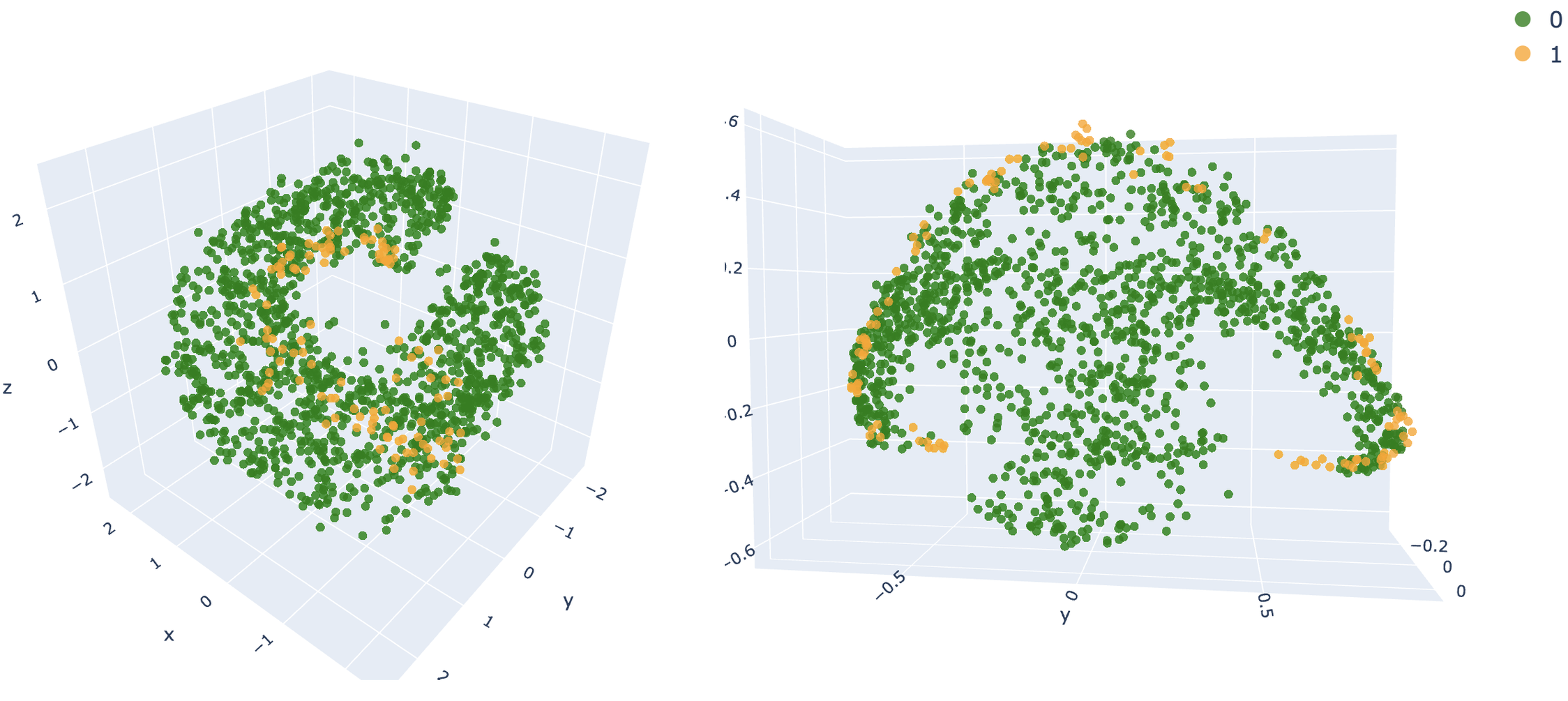

Supplement: S3 Fig — Comparison of the latent representations of the model E1M2, trained at high capacity (left) and low capacity (right), in three dimensions. (TIFF) [file pcbi.1012952.s003.tif]

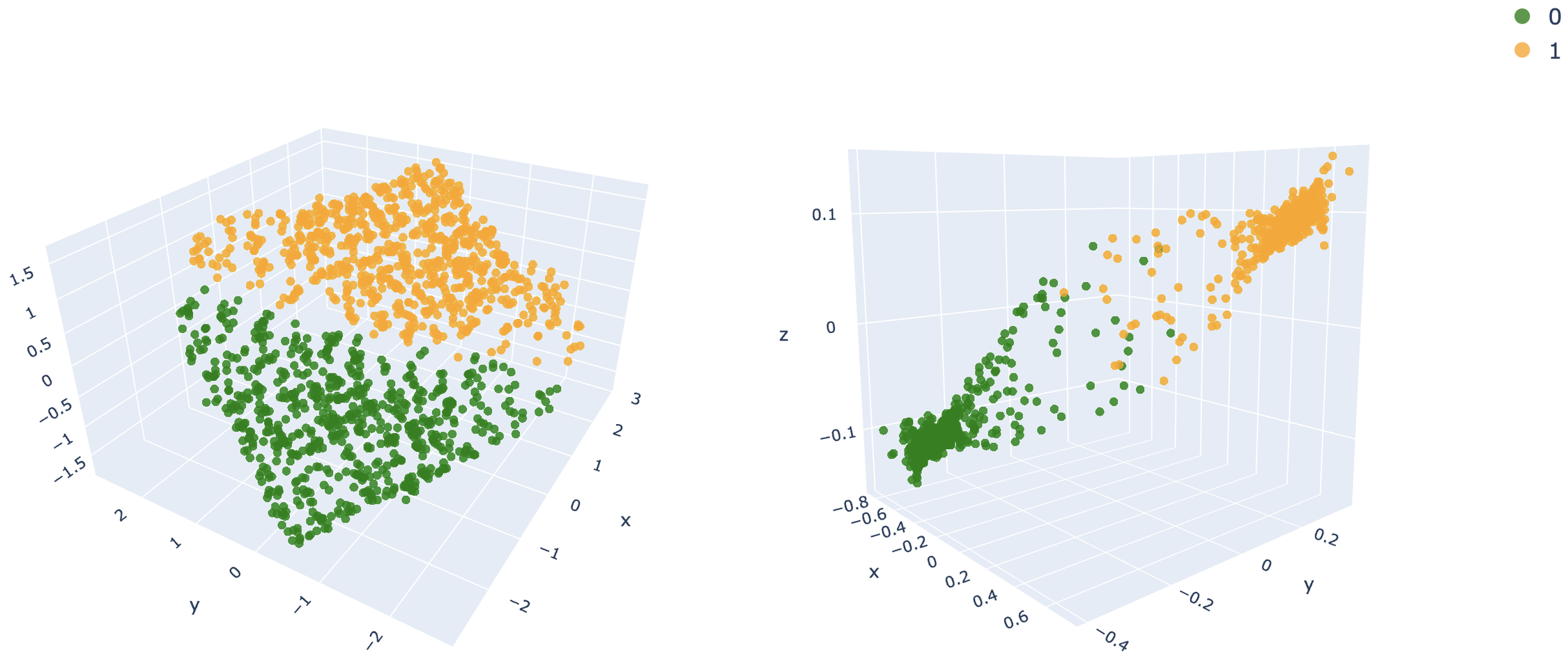

Supplement: S4 Fig — Comparison of the latent representations of the model E2M1, trained at high capacity (left) and low capacity (right), in three dimensions. (TIFF) [file pcbi.1012952.s004.tif]

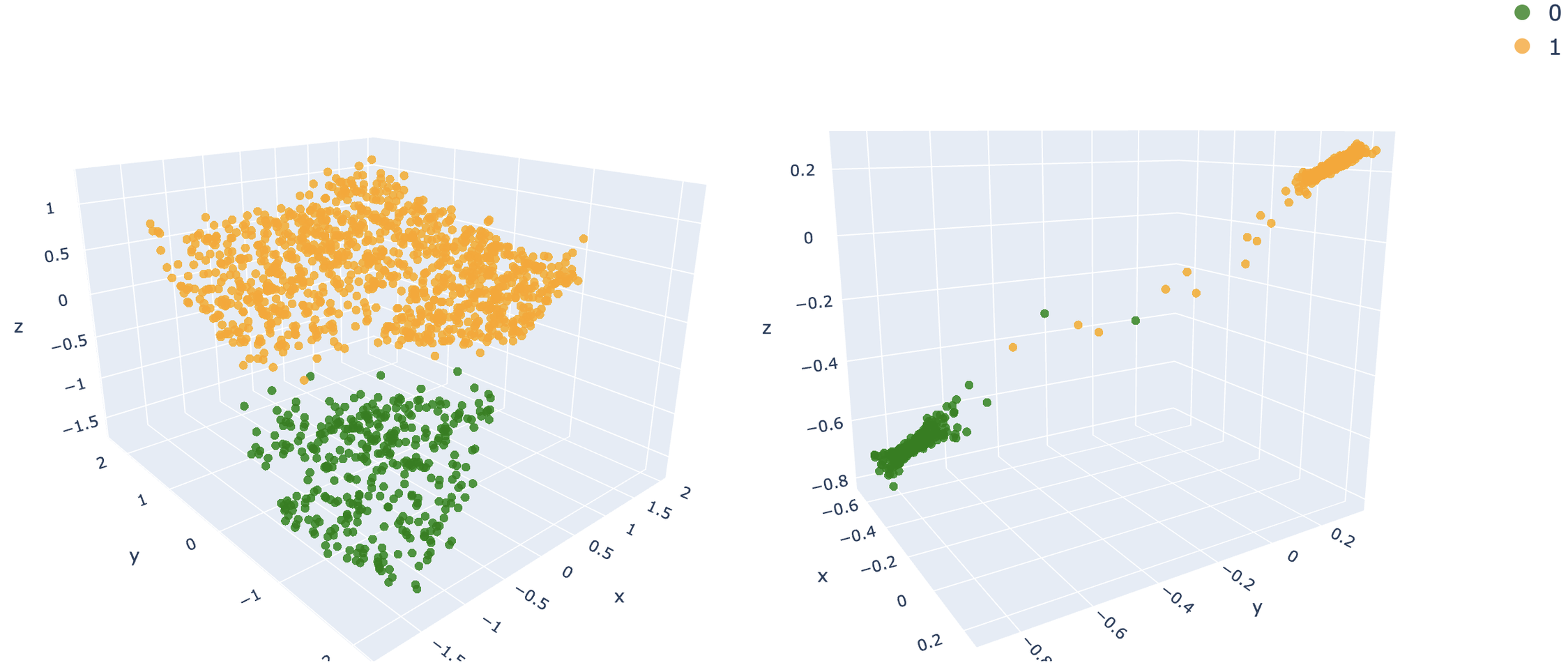

Supplement: S5 Fig — Comparison of the latent representations of the model E2M2, trained at high capacity (left) and low capacity (right), in three dimensions. (TIFF) [file pcbi.1012952.s005.tif]

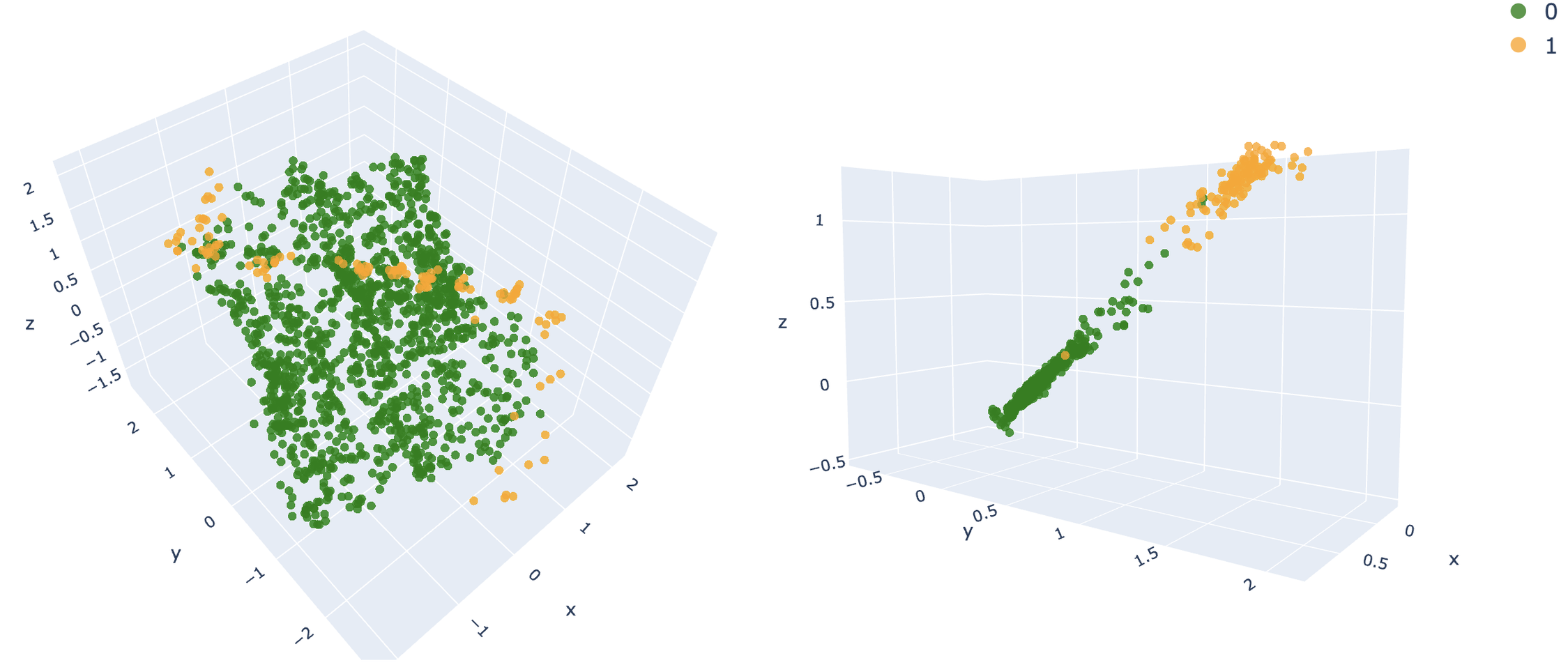

Supplement: S6 Fig — Comparison of the latent representations of the model E2M3, trained at high capacity (left) and low capacity (right), in three dimensions. (TIFF) [file pcbi.1012952.s006.tif]

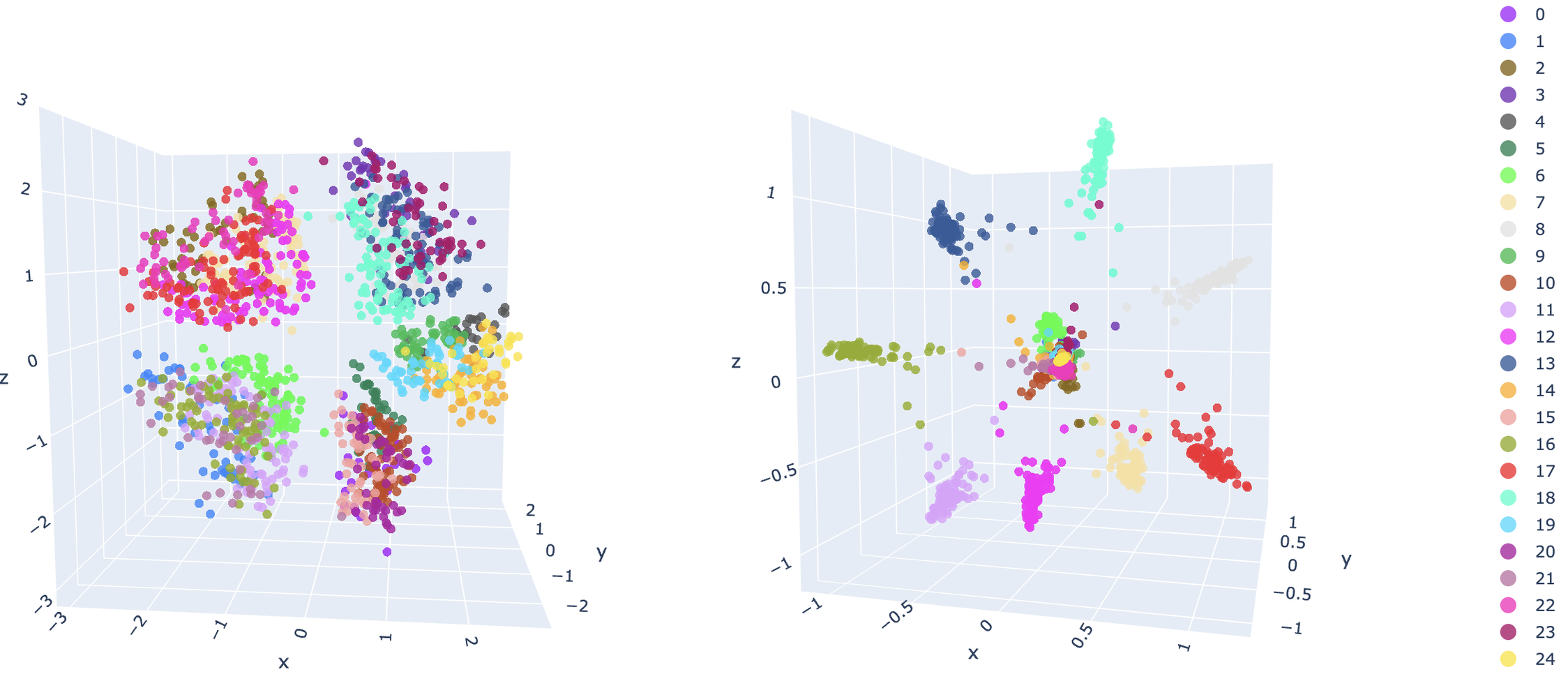

Supplement: S7 Fig — Comparison of the latent representations of the model E2M4, trained at high capacity (left) and low capacity (right), in three dimensions. (TIFF) [file pcbi.1012952.s007.tif]

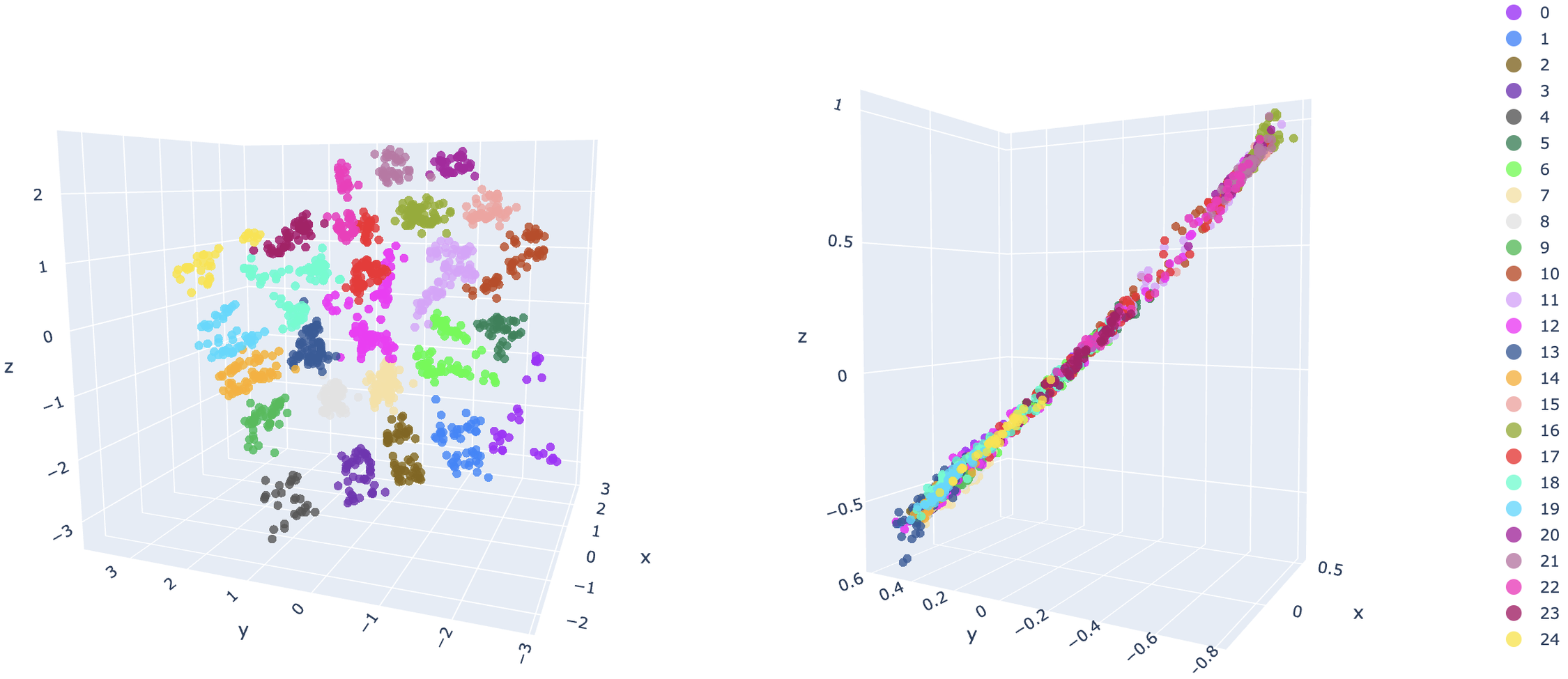

Supplement: S8 Fig — Comparison of the latent representations of the model E2M5, trained at high capacity (left) and low capacity (right), in three dimensions. (TIFF) [file pcbi.1012952.s008.tif]
